# PSO-Based Algorithm Applied to Quadcopter Micro Air Vehicle Controller Design

**DOI:** 10.3390/mi7090168

**Published:** 2016-09-15

**Authors:** Huu-Khoa Tran, Juing-Shian Chiou

**Affiliations:** 1 Department for Management of Science and Technology Development, Ton Duc Thang University, Ho Chi Minh City, Vietnam; tranhuukhoa@tdt.edu.vn; 2 Faculty of Electrical & Electronic Engineering, Ton Duc Thang University, Ho Chi Minh City, Vietnam; 3 Department of Electrical Engineering, Southern Taiwan University of Science and Technology, Tainan 71005, Taiwan

**Keywords:** particle swarm optimization (PSO)-based, evolutionary programming (EP), integral of the squared error (ISE), micro air vehicle (MAV)

## Abstract

Due to the rapid development of science and technology in recent times, many effective controllers are designed and applied successfully to complicated systems. The significant task of controller design is to determine optimized control gains in a short period of time. With this purpose in mind, a combination of the particle swarm optimization (PSO)-based algorithm and the evolutionary programming (EP) algorithm is introduced in this article. The benefit of this integration algorithm is the creation of new best-parameters for control design schemes. The proposed controller designs are then demonstrated to have the best performance for nonlinear micro air vehicle models.

## 1. Introduction

The state of the art design first developed by Kennedy and Eberhart [1] in 1995 is particle swarm optimization (PSO)-based on inspiration from the social behavior of groups of birds and fish. The PSO-based algorithm is effortless and simply requires the adjustment of a few parameters [2]. Additionally, when compared with other methods, such as neural networks, machine learning, and genetic computation, it achieves better performances in computing speed, accuracy, and small memory size. The advantages of the PSO-based algorithm are very impressive, and as a result, many scientists have used it to solve their problems, ranging from shop-scheduling to optimized control gains. Some others have even upgraded this algorithm by combining PSO with another methodology. Hence, different types of hybrid PSOs have been proposed due to multi-objective functioning. For example, Liu et al. [3] used a PSO-based memetic algorithm (PSOMA) to solve a flow shops scheduling issue. Later, Solihin et al., using a PSO-based algorithm, optimized the state feedback for a flexible link manipulator tracking control [4]. The combination of PSO and gravitational search algorithm (GSA) was introduced by Mirjalili et al. [5] in 2010. A novel hybrid binary particle swarm optimization algorithm (HBPSO) that combined the PSO’s concept and a Genetic Algorithm (GA) was presented by Menhas et al. [6]. Ghodrati and Lotfi combined cuckoo search (CS) and particle swarm optimization (PSO) [7] in 2012. A hybrid particle swarm optimization algorithm (HPSOM) used to integrate the PSO algorithm with the genetic algorithm mutation method was introduced by Esmin and Matwin [8]. A PSO-based algorithm was also used by Koyuncua and Erolb [9] to cope with scheduling new product development projects in 2015. All of these methods stated above can increase the convergence speed and improve the chosen system. Following this trend, this research paper focuses on the PSO-based algorithm and upgrades this algorithm by using evolutionary programming [10]. Its benefit is that it creates new best-parameters in a short time, improving the multi-object optimization process.

Conventional proportional-integral-derivative (PID) controllers that have a simple control structure are applied in many industrial fields. Moreover, due to regulated control gains, a new hybrid control approach fuzzy-PID [11,12] is applied for an autonomous mini-helicopter. In this paper, a fuzzy-PID controller [11,12,13] that has various advantages is chosen for the controller design task. The parameter gains are then evaluated by using the fitness functions. The Integral of the Squared Error (ISE) [14] that is associated with the system’s performance indices is selected for this assignment. The attitude-based models of the quadcopter micro air vehicle are exploited to illustrate the augmenting benefits.

## 2. PSO-Based Algorithm Applied Controller Design

Evolutionary algorithms are a group of algorithms that include genetic algorithm (GA) and evolutionary programming (EP). Although similar to genetic programming (GP), the EP method allows for the evolution of the optimized parameters and having a fixed structure. The EP process neglects the crossover operator while keeping the main operator mutation and the selection strategy. The populations’ members are observed as a part of a specific species rather than similar species members; thus, a new offspring is generated by each parent [10].

The mutation stage occurs at every *x_i_*(*t*) and generates *X_j_*(*t*) = [*x_j_*_1_(*t*), *x_j_*_2_(*t*), … , *x_j_*_D_(*t*)] by using Equation (1):

(1) $$x_{jD}(t+1)=x_{jD}(t)+N\left(0,1\right)$$

where *x_j_*_D_ is the *j*th individual error data, and *N*(0,1) is a random normal distribution of *x*. The mutation rate is noted as *mr*.

The next step is the selection operator wherein the new individual of every particle *j* is selected by using the roulette wheel technique. It picks *x_j_*_D_ from the set of all *X_j_*, then updates *v_i_* and *x_i_* by the major equation of the PSO-based algorithm. In this article, the proposed algorithm creates an elite PSO-based algorithm by the combination of PSO and EP. These effects usually attain a better result than either the PSO or the existing algorithms alone.

The fundamental PSO algorithm is a great number of particles moving around in a multi-dimensional space, such as the schooling of fish, the flocking of birds, and the swarm theory [1,2]. The major PSO algorithm can be verified by Equation (2):

(2) $$\left\{ \begin{array}{l} v_{iD}(t+1)=\omega \times v_{iD}(t)+l_{1}\times {\mathrm{rand}}_{1}(\cdot )\times \left(p_{iD}(t)-x_{iD}(t)\right)+l_{2}\times {\mathrm{rand}}_{2}(\cdot )\times \left(p_{gD}(t)-x_{iD}(t)\right)\\ x_{iD}(t+1)=x_{iD}(t)+v_{iD}(t+1) \end{array}\right.$$

The velocity and the position of particle *i* are noted by *v_i_*_D_ and *x_i_*_D_, respectively. The best particle historical position is *p*_*i*__D_, and the global best position is *p_g_*_D_. In order to go along with learning rates *l*_1_ and *l*_2_, the inertia weight ω is a user-defined parameter. It manages the relationship of the previous values of particle velocities to the current value. The rand_1_(·), rand_2_(·) items are uniformly distributed random numbers [0,1]. The *l*_1_ × rand_1_(·) × (*p_i_*_D_(*t*) − *x_i_*_D_(*t*)) term refers to the cognitive component. It reflects the distance at which the best solution *P_i_*(*t*) of a particle is located. The combination of the PSO-based algorithm and EP will generate and update the parameters of the control system performance index. Some specific performance indicators are usually designed to evaluate and determine the minimum error criterion [14]. Due to system advantages, the ISE performance index is chosen as shown in Equation (3):

(3) $$\mathrm{ISE}=\int_{0}^{\tau }e{\left(t\right)}^{2}dt$$

The performance index *f*(ISE) is the minima for all swarm particles, meaning that the optimization issue determines a group of five fuzzy-PID control parameters *k*_P_, *k*_I_, *k*_D_, *k_e_*, and *k_de_*. After that, each new particle is said to represent a group of solutions. The four items rising time, settling time, peak time, and maximum overshoot are of significant focus on each control system. They are exploited to find out the minimum ISE fitness function, as shown in Equation (4).

(4) $$f(\mathrm{ISE})=\alpha_{1}\times \mathrm{RiseTime}+\alpha_{2}\times \mathrm{SettlingTime}+\alpha_{3}\times \mathrm{PeakTime}+\alpha_{4}\times (|r-\mathrm{Overshoot}|)$$

The weighting factors of each control factor are noted as α*_i_*. The rising time, settling time, peak time, and maximum overshoot are estimated via the output performance, and then its values are recorded. Afterward, the particle groups which contain a large error can be eliminated. Thus, the convergence speed of the system is also accelerated. The fitness function settles in the range:

f(ISE)∈[0,100]

**Figure 1:**
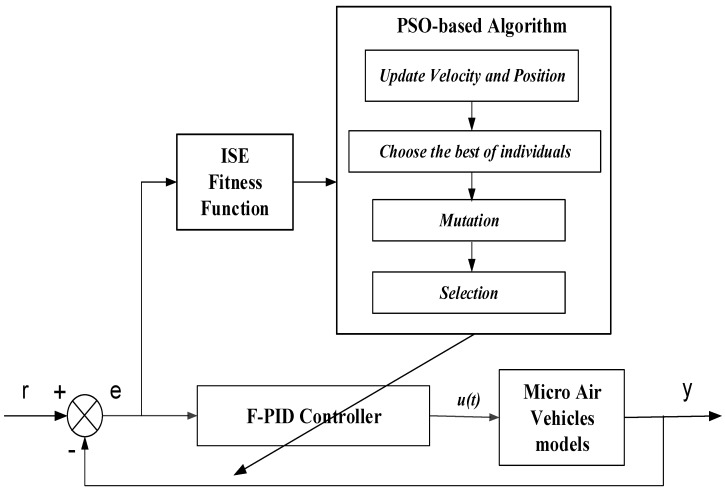
Particle swarm optimization based (PSO)-based algorithm applies to the micro air vehicle controller design.

**Figure 2:**
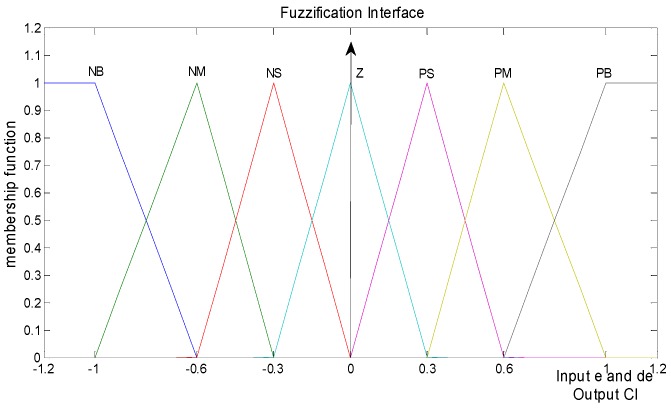
Inputs *e* and *de*, and output CI of Fuzzification interface.

The whole progression can be assigned as illustrated in Figure 1:

The main functions of PID controllers are improving the dynamic response and reducing or eliminating the steady state errors. The improvement of the fuzzy-PID controller by incorporating a fast learning PID controller gains with fuzzy control parameters, yielding a high-quality solution [15], is chosen in this article. As shown in Figure 2, the tracking error *e*(*t*) and the differential tracking error *de*(*t*) after modifying the triangle membership function in segments (0, 0.3, 0.6, and 1) are the inputs of the fuzzy inference system. The two inputs *e*(*t*) and *de*(*t*), and the output CI are formal triangular membership functions.

Based on expert knowledge, the dynamic behavior of the fuzzy logic controller (FLC) is described by a set of linguistic rules [16]. In this paper, we employed Mandani’s Min–Max inference engine type and center of area method (COA) defuzzification. Seven partitions are decomposed and implemented to fuzzy control parameters: negative big (NB), negative medium (NM), negative small (NS), zero (ZE), positive small (PS), positive medium (PM) and positive big (PB). The application of fuzzy control rules that have only three NB and three PB are then designated in Table 1. The structure of the fuzzy-PID input is presented by Equation (5).

(5) $$u(t)=u_{\mathrm{Fuzzy}}+u_{\mathrm{PID}}=u_{\mathrm{Fuzzy}}+k_{P}\times e+k_{I}\int e+k_{D}\times \frac{de}{dt}$$

## 3. Quadcopter as a Micro Air Vehicle

**Figure 3:**
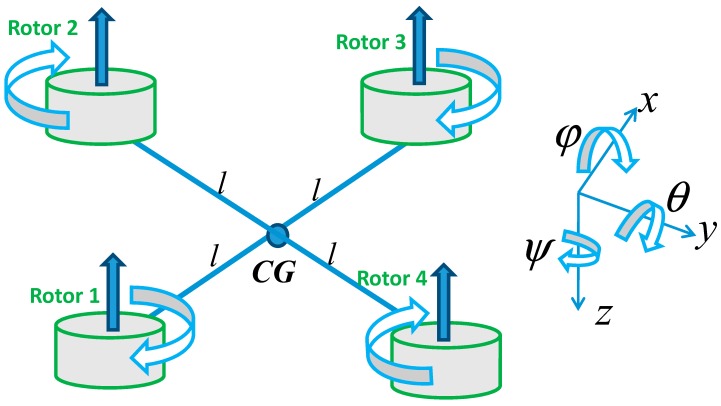
The micro air vehicle quadcopter model.

The quadcopter as a micro air vehicle (MAV) [17,18] in Figure 3 has six degrees of freedom (DOF) and is depicted using a right hand generalized Earth coordinate system of axes and a right hand body frame. It has a special form with two pairs of contra-rotating rotors to provide lift and directional control. Unlike conventional helicopters, it typically has fixed-pitch blades and varies their thrust by changing rotor speeds. The quadcopter has two major motivating benefits, which are its reliability and compactness. The Quadcopter configuration can be described as having four propellers, with two pairs of propellers (1 and 3) and (2 and 4) that turn in opposite directions in a cross configuration. By varying the rotor speed, the lift force and the motion-creation are changed. Hence, vertical motion is generated by increasing or decreasing the four propeller’s speeds simultaneously. Changing the speed of propellers 2 and 4 conversely produces roll rotation that goes together with lateral motion. Pitch rotation and the corresponding lateral motion is the result of the speed of propellers 1 and 3 being conversely modified. As it results from the difference in the counter-torque between each pair of propellers, yaw rotation is more subtle.

## 4. Simulation Results

The previously mentioned parameters are established for the numerical simulation test in this section. The PSO-based algorithm for attitude pilot angle: Roll, Pitch, and Yaw are set on 30, 50, and 20 generations, respectively. The fitness weighting factors that occurred in Equation (4) are set as [α_1_, α_2_, α_3_, α_4_] = [40, 25, 10, 5], and PID gains are set in the range [0, 20]. The mutation rate is *mr* = 0.105. The attitude control performances are displayed in each channel: Roll angle is set 1 rad (~60°), Pitch angle is set 0.5 rad (~30°) and Yaw angle is set 1 rad (~60°). The sampling time in this simulation is 0.01 s.

The referential transfer functions that applied to controller designs are derived from [15,16]:

Roll channel (Lateral control):

$$f(s)=\frac{0.522}{0.004s^{4}+0.039s^{3}+0.009s^{2}}$$

Pitch channel (Longitudinal control):

$$\theta (s)=\frac{0.522}{0.004s^{4}+0.039s^{3}+0.009s^{2}}$$

Yaw channel (Pedal control):

$$\psi (s)=\frac{21.78}{0.008s^{4}+0.077s^{3}+0.18s^{2}}$$

**Figure 4:**
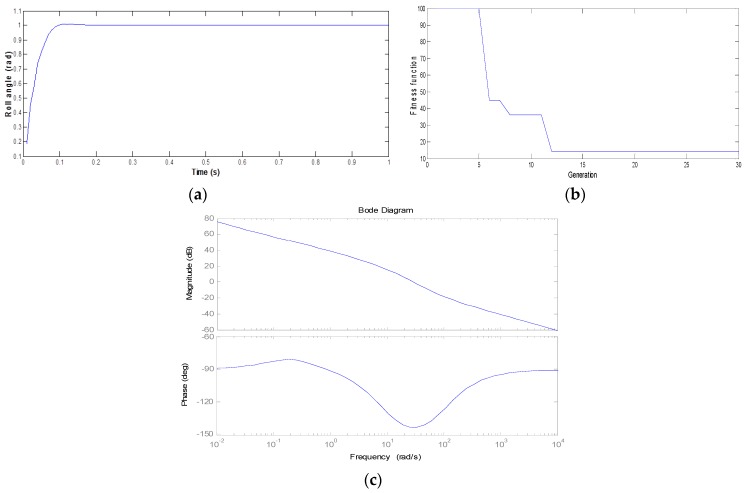
Roll channel control with the PSO-based algorithm. (**a**) Roll angle response; (**b**) The ISE fitness function; (**c**) Bode diagram.

**Figure 5:**
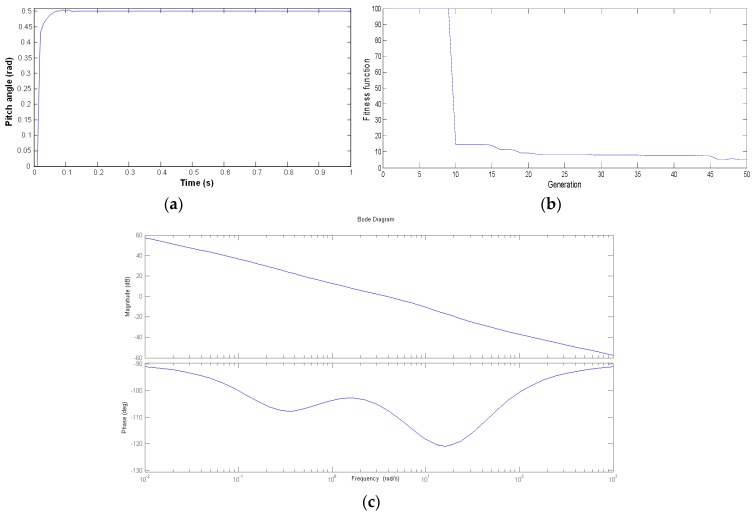
Pitch channel control with the PSO-based algorithm. (**a**) Pitch angle response; (**b**) The ISE fitness function; (**c**) Bode diagram.

**Figure 6:**
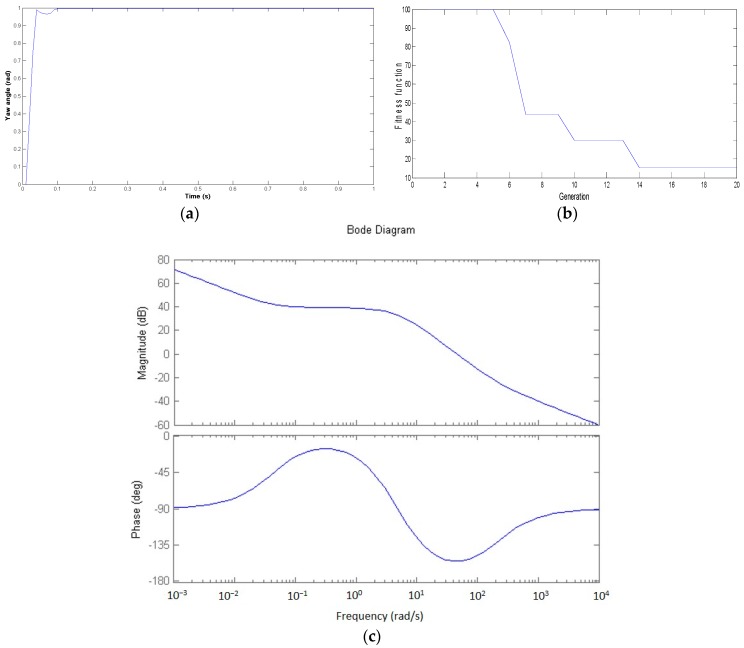
Yaw channel control with the PSO-based algorithm. (**a**)Yaw angle response; (**b**) The ISE fitness function; (**c**) Bode diagram.

The results of the PSO-based controller strategy that are shown in Figure 4, Figure 5 and Figure 6 are examined. After using the proposed controller methodology, the Bode diagrams have shown on each channel Roll, Pitch, and Yaw results that are within the stability margin of the system. The convergence speed of the PSO-based algorithm is rapid with success after just 15 iterations for the overall process. In addition, the impressive results of the attitude pilot responses are achieved after 0.2 s.

## 5. Conclusions

In this article, the proposed PSO-based algorithm—which optimized fuzzy-PID controller gains and achieved minimization by the ISE fitness criterion—has been successfully implemented to attitude control Roll, Pitch, and Yaw of the quadcopter micro air vehicle models. The best performances are successfully accomplished by implementing the proposed controller. The algorithm saves the settling time and improves the reliability, as well as the stability of the system models. Due to demonstration of the augmented benefits of PSO algorithm, real time flight control is to be future research work.

## Figures and Tables

**Table 1:** The Fuzzy rule-table.

CI(*t*)		*e*(*t*)						
		NB	NM	NS	ZE	PS	PM	PB
de(t)	NB	ZE	NS	NS	NM	NM	NB	NB
	NM	PS	ZE	NS	NS	NM	NM	NB
	NS	PS	PS	ZE	NS	NS	NM	NM
	ZE	PS	NM	PS	ZE	NS	NS	NM
	PS	PM	PM	PS	PS	ZE	NS	NS
	PM	PB	PM	PM	PS	PS	ZE	NS
	PB	PB	PB	PM	PM	PS	PS	ZE

NB: negative big; NM: negative medium; NS: negative small; ZE: zero; PS: positive small; PM: positive medium; and PB: positive big.
